# *E. coli* Enterotoxin LtB Enhances Vaccine-Induced Anti-*H. pylori* Protection by Promoting Leukocyte Migration into Gastric Mucus via Inflammatory Lesions

**DOI:** 10.3390/cells8090982

**Published:** 2019-08-27

**Authors:** Xiaoyan Peng, Rongguang Zhang, Chen Wang, Feiyan Yu, Mingyang Yu, Shuaiyin Chen, Qingtang Fan, Yuanlin Xi, Guangcai Duan

**Affiliations:** 1 Department of Epidemiology and Statistics, College of Public Health, Zhengzhou University, Zhengzhou 450001, China; 2 Department of Basic Medicine, Chuxiong Medical College, Chuxiong 675005, China

**Keywords:** *Helicobactor pylori*, gastric mucus, *Lactococcus lactis*, LtB, protective immunity, Th17

## Abstract

Current studies indicate that the anti-*H. pylori* protective efficacy of oral vaccines to a large extent depends on using mucosal adjuvants like *E. coli* heat-lable enterotoxin B unit (LtB). However, the mechanism by which Th17/Th1-driven cellular immunity kills *H. pylori* and the role of LtB remains unclear. Here, two *L.* *lactis* strains, expressing *H. pylori* NapA and LtB, respectively, were orally administrated to mice. As observed, the administration of LtB significantly enhanced the fecal SIgA level and decreased gastric *H. pylori* colonization, but also markedly aggravated gastric inflammatory injury. Both NapA group and NapA+LtB group had elevated splenocyte production of IL-8, IL-10, IL-12, IL-17, IL-23 and INF-γ. Notably, gastric leukocytes’ migration or leakage into the mucus was observed more frequently in NapA+LtB group than in NapA group. This report is the first that discusses how LtB enhances vaccine-induced anti-*H. pylori* efficacy by aggravating gastric injury and leukocytes’ movement into the mucus layer. Significantly, it brings up a novel explanation for the mechanism underlying mucosal cellular immunity destroying the non-invasive pathogens. More importantly, the findings suggest the necessity to further evaluate LtB’s potential hazards to humans before extending its applications. Thus, this report can provide considerable impact on the fields of mucosal immunology and vaccinology.

## 1. Introduction

Mucosal infections are worldwide public health threats. Accompanying increasing antibiotic resistance, safe, effective, and affordable vaccination strategies have become more urgently needed than ever [1,2]. As for gastrointestinal pathogens, oral vaccination may block infection at their entries, making it more attractive than other prevention approaches [3]. However, there aren’t any oral vaccines that are commercially available for most of the predominantly non-invasive infections such as *Helicobacter pylori* and enterotoxigenic *Escherichia coli* (ETEC) [3,4]. The reasons for this include lacking an effective immune adjuvant, delivery vehicles, and especially knowledge of the immune protection mechanism [4].

Firstly, the mechanism underlying vaccine-induced protection against *H. pylori* is still poorly understood, which is an unavoidable constraint and challenge to vaccine development [4,5]. In the early stage, the protective effect was ascribed to increased SIgA and Th2 responses induced by oral vaccination, in part owing to *H. pylori* colonization mainly in gastric mucus layer [6]. Then the experiments on B-cell-deficient and IL-5-deficient mice proved independence of the immune protection from any antibodies and Th2 response [7,8]. Certain reports pointed that elevated mucosal SIgA and Th1 responses in natural infection with *H. pylori* commonly result in aggravated gastritis and gastric injury but no protective effects, and the vaccine-induced protection depends on a mixed Th1/Th2 but not antibody responses [9]. Currently, most data support the idea that the anti-challenge protection is mainly mediated by the Th1/Th17 polarized responses, and the Th1 might take the role of Th17 in the absence of a Th17 response [10,11].

However, supposing that the anti-*H. pylori* protection is assuredly mediated by Th1/Th17 responses, an inevitable problem we have to face is how the immune effector cells act on this non-invasive bacterium, which mainly colonizes the gastric mucus layer. The mechanism of immune system protection against *H. pylori* might be an immunization model for many mucosal non-invasive infections. Thus, addressing this problem is significant for the development of mucosal immunology.

Secondly, accumulating evidence shows that the protective efficacy of oral vaccination to a large degree depends on the mucosal adjuvants used in combination with vaccine antigens. Currently, certain bacterial toxins, such as *E. coli* heat-labile toxin (LT), cholera toxin (CT), shiga-like toxin (SLT) and their derivatives, have proved to be potent immune adjuvants [12]. In particular, LT subunit B (LtB) has been extensively used in animals and even clinical trials because it is generally deemed as being nontoxic for being free of enzymatic activity [13,14,15]. Nevertheless, the accurate role of LtB in the immune protection is undefined. Most studies have put foci on LtB’s activity to enhance immunity, but rarely on the potential unbeneficial effects. Under the present situation, extending LtB application in clinical trials may bring a high risk of harm to the recipients.

Thirdly, in order to deliver antigens to mucosal sites, attenuated pathogenic bacteria, viruses, fungi, probiotics, plants, and nonliving carriers like nanoparticles have been exploited as vaccine vehicle candidates [16,17,18]. Among them, probiotics have showed considerable benefits with the capacity of biosynthesizing, delivering and protecting vaccine antigens, and the adjuvantivity and safety for human use [19,20]. *L. lactis,* a typical probiotic bacterium used in food processing with long history, has been thought to be a promising oral vaccine delivery vector [19,21]. As reported, oral delivery of papillomavirus 16 E7 antigen by *L. lactis* protected the mice from infection caused by the virus challenges [21]. As for anti-*H. pylori* immunization, however, most studies showed oral gavages with *L. lactis*-delivered *H. pylori* antigens could induce elevated systemic and mucosal immune responses, or reduce gastric bacterial burden, but would rarely be able to prevent *H. pylori* infection, suggesting the relatively weak adjuvant effect of *L. lactis* and the necessity of using additional adjuvant in the *L. lactis*-vectored *H. pylori* vaccines [19,22,23].

*H. pylori* neutrophil-activating protein subunit A (NapA) can mediate bacterial binding to gastric epithelium via adherence to carbohydrates, stimulate the epithelial cells to produce interleukin (IL)-8, activate neutrophils and dentritic cells by the toll-like receptor 2 (TLR2), and participate in pathogenesis [24]. Meanwhile, NapA has shown protective antigenicity, capacity for promoting Th1/Th17-biased polarization, and is universally thought as a promising vaccine candidate and immune modulatory agent [25,26]. These data warrant using NapA as a model of vaccine antigens to investigate the mechanism of LtB-adjuvanted vaccine-evoked immune protection.

Here, an engineered *L. lactis* strain expressing NapA alone or in conjunction with LtB was orally administrated to mice, the anti-*H. pylori* protective efficacy, immune response profiles and gastric histological changes post-immunization and bacterial challenges were evaluated, and the possible immune mechanism and the role of LtB were discussed. A novel explanation was proposed herein for the mechanism beneath the LtB-assisted oral vaccine-induced cellular immunity against *L. lactis*. Meanwhile, the report is the first to display the serious problem on safety related to the application of LtB in humans, which may have a critical impact on future research.

## 2. Materials and Methods

### 2.1. Bacteria and Cultivation

The bacterial strains and plasmids are listed in Table S1 (Supplementary Materials). *L. lactis*, *H. pylori* and *E. coli* were cultivated using GM17 medium, sheep blood containing Brucella agar plates and Luria Broth medium, respectively, as reported previously [27,28].

### 2.2. L. lactis Production of NapA and LtB, Respectively

The engineered *L. lactis* strains, NZ3900/pNZ-*sp*-*ltB* and NZ3900/pNZ-*∆sp*-*napA*, were cultivated, respectively, using GM17 media at 30 °C, 5% CO_2_. When OD600 of the culture ≈ 0.35, induction of NapA expression was started by adding nisin at a final concentration of 40 μg/L to the culture, which was maintained for 5 h. *L. lactis* cell lysate samples were prepared via lysozyme digestion and supersonic sonication, and analyzed through SDS-PAGE and western-blot assays using mouse anti-*H. pylori* sera and mouse anti-LTB antibody (Abcam, Shanghai, China), respectively, as the primary antibody [27,29]. The supernatant of the bacterial culture was sampled and pretreated for SDS-PAGE using the trichloroacetic acid precipitation method reported previously [22].

### 2.3. Oral Vaccination of Mice

**Figure 1:**
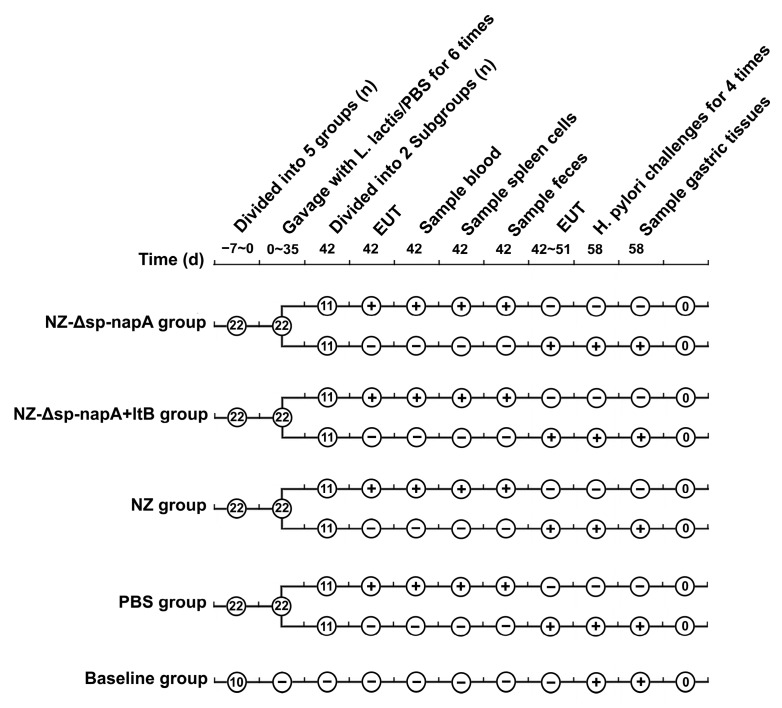
Experimental management of the BALB/c mice. +: received the treatment; −: was not given the treatment; EUT: euthanasia.

The project has been approved by Zhengzhou University Institutional Review Board, and complied with the ARRIVE guidelines. SPF BALB/c mice, 6 w old, were supplied by Henan Experimental Animal Center (Zhengzhou, China). The mice were randomly assigned to 4 groups and treated as shown in Figure 1. The mice of NZ-Δsp-*napA*, NZ-Δsp-*napA*+*ltB*, NZ and PBS groups, 22 mice each, were orally gavaged with bacterial suspension of *L. lactis* NZ3900/pNZ-Δsp-*napA* (5 × 10^10^ CFU/mL), a mixture of NZ3900/pNZ-Δsp-*napA* (5 × 10^10^ CFU/mL) and NZ3900/pNZ-sp-*ltB* (5 × 10^10^ CFU/mL), NZ3900/pNZ8110 (5 × 10^10^ CFU/mL), and PBS, respectively, with 250 μL for each mouse, on day 0, 7, 14, 21, 28, and 35. The mice were deprived of food and water for the gavages as described [25]. The Baseline group (*n* = 10) did not receive any gavages. For quality control of the vaccines, SDS-PAGE analysis was performed to ensure NapA/LtB expression in the *L. lactis* strains prior to the vaccinations.

### 2.4. Sampling Blood, Spleen and Intestinal Feces

Blood, spleen and intestinal feces of the mice were sampled using the methods reported previously [22,25]. Briefly, seven days after the last vaccination, half of the gavaged mice were taken blood from their orbital sinus, and then slaughtered by cervical dislocation. After soaking the mice in 75% (*v/v*) alcohol for 3 min, the mice were dissected. The spleens were separated and used for splenocyte cultivation as described below. Intestinal feces of the mice were collected by injecting 1 mL PBS containing 0.1 mM phenylmethanesulfonyl fluoride into the intestinal lumen. The feces suspension was stored at 4 °C for about 14 h, then the supernatant was separated via centrifugation, and used in ELISA testing for fecal SIgA.

### 2.5. ELISA Detection of IgG and SIgA Antibodies

To prepare the detector antigen for ELISA detection of NapA-specific IgG and SIgA antibodies, the previously engineered *E. coli* strain TB1/pMAL-c2x-linker-*napA* was cultured and induced using isopropyl-β-d-thiogalactoside (IPTG), and the recombinant NapA (rNapA) in fusion with maltose binding protein was purified by amylose affinity chromatography as instructed (NEB, Ltd., Ipswich, MA, USA) [30]. The specific serum IgG and fecal SIgA antibodies were quantified by ELISA with the purified rNapA as the detector antigen [25,30]. The absorbance of each well at 450 nm (OD_450_) was measured and designated as indicators of the specific SIgA and IgG levels.

### 2.6. Splenocyte Cultivation and Antigenic Stimulation

Splenocyte cultivation and antigenic stimulation were performed as described before [25]. In brief, the spleens of mice were homogenized, and the splenocytes were screened by passing a 20 mesh strainer, erythrocyte lysing, washing and suspending with D-hanks solution. Then splenocytes were resuspended in RPMI-1640 medium, and the cellular suspension was added to culture plates. To stimulate production of cytokines, *H. pylori* cell lysates were added to the cellular cultures. The cultivation was carried out at 37 °C, 5% CO_2_ in a cell incubator for 72 h. These experiments were replicated in three wells of the cell culture plates for each mouse.

### 2.7. Assessment of Cytokines

On day 3 of splenocyte cultivation, the culture was centrifugated at 3000 rpm for 20 min. The supernatant of the culture was separated and used for assessment of cytokine production levels of the splenocytes upon stimulation with *H. pylori* antigens. To distinguish Th1, Th2 and Th17, concentrations of interferon (IFN)-γ, interleukin (IL)-2, IL-4, IL-8, IL-10, IL-12, IL-17, and IL-23 were determined using ELISA kits (Mlbio, Shanghai, China), with reference to the product description.

### 2.8. H. pylori Challenges

*H. pylori* 11637 was cultivated with sheep blood-containing Brucella agar plates at 37 ℃, 10% CO2, 85% N2 and 5% O2, for approximate 3 days [28]. Then the bacteria were harvested from culture plates, suspended with the culture medium, and the concentration of the bacterial suspension was adjusted to be 1 × 10^10^ CFU/mL. Except the Baseline group, all the mice were gavaged with *H. pylori* suspension (200 μL each) on day 7, 10, 13, and 16 post-immunization as described elsewhere [25].

### 2.9. Evaluation of H. pylori Colonization

On day 7 after the H. pylori challenges, all the mice were sacrificed, two round pieces of the gastric wall were dissected from the gastric pyloric antrum using a puncher for each mouse, homogenized on a stainless strainer of 200 mesh and washed using *H. pylori* preservation fluid of 1 mL (100 g/L sucrose, 500 mL/L fetal bovine serum), and the filtrate was tested by urease activity assays and bacterial cultivation as reported [25,28]. The mice of Baseline group were used for determining the gastric urease activity and *H. pylori* colonization of the unintervened mice used in this study.

### 2.10. Gastric Histological Examination

Histological examination was performed to evaluate gastric inflammatory changes of the mice. The stomach tissues were fixed using 10% formaldehyde solution, and examined by paraffin section and hematoxylin-eosin staining. Gastric inflammatory responses of the mice were graded using the reported method [25,31].

### 2.11. Statistical Analysis

The data were presented as means ± standard deviation (*x* ± *s*), and compared by one-way variance analysis using SPSS 21.0. The least significant deviation method (LSD) was used for pairwise comparisons. The difference was inferred as significant at *p* < 0.05. 

## 3. Results

### 3.1. Cultivation of the Engineered L. lactis Strains

**Figure 2:**
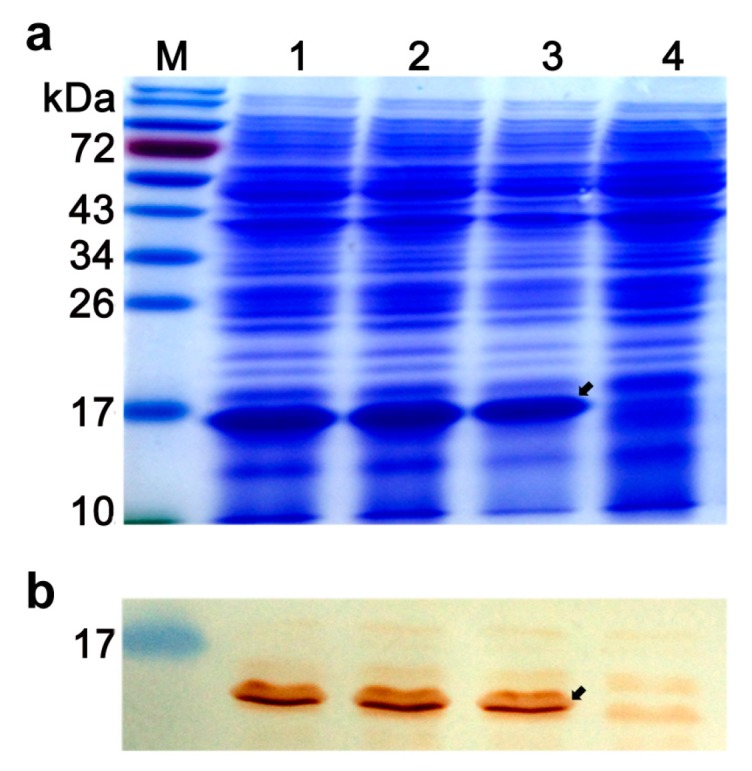
SDS-PAGE (**a**) and western-blot assays (**b**) of cellular lysates of nisin-induced *L. lactis* strains. Mouse anti-*H. pylori* sera were obtained by subcutaneous immunization with *H. pylori* lysates plus Freund’s adjuvant, and used as the primary antibody. 1,2,3, *L. lactis* NZ3900/pNZ-*Δsp-napA*; 4, cell lysates of NZ3900/pNZ8110. Arrows indicate NapA expression products. Appearance of positive reaction band at approximately16 kDa demonstrated antigenicity of the *napA* expression product.

The engineered *L. lactis* strains were induced with nisin for expression of the heterogenous proteins. SDS-PAGE showed the expression products of NapA and LtB were 16 kDa and 13 kDa, respectively. The NapA product constituted 14% of the cell lysate proteins, while LtB occupied 10% of the supernatant proteins of the bacterial culture. Western blotting analysis showed that the NapA and LtB were able to be recognized by the mouse anti-*H. pylori* sera and mouse anti-LtB antibody (Abcam, Shanghai, China) (Figure 2). The observations confirmed the efficient expression and immune activity of the recombinant proteins.

### 3.2. Immunization of Mice and Antibody Assays

**Figure 3:**
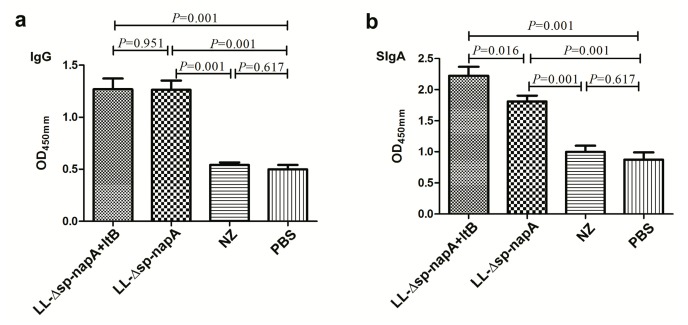
Evaluation of anti-NapA IgG and SIgA levels of the mice. (**a**) ELISA results of serum anti-NapA IgG antibody. (**b**) ELISA data of fecal NapA-specific SIgA antibody. The NZ-*Δsp-napA*, NZ-*Δsp-napA+ltB*, NZ and PBS groups (n = 11 each) were orally gavaged with bacterial suspension of NZ3900/pNZ-Δsp-*napA*, mixture of NZ3900/pNZ-Δsp-*napA* and NZ3900/pNZ-sp-*ltB*, NZ3900/pNZ8110, PBS, respectively. The sera and feces of mice were sampled from the mice one week after the immunization, diluted at 1:50 and tested for anti-NapA immunoglobulins. ELISA tests were replicated for three times. Data were shown as mean ± SEM. Error bars indicate standard deviations.

To prepare the detector antigen for ELISA detection of NapA-specific antibodies, another recombinant NapA, expressed in fusion with maltose binding protein, was purified from *E. coli* TB1/pMAL-c2x-*linker*-*napA*. The purified fusion protein (rNapA) had a molecular weight of approximately 57 kDa and 90% purity, capable of being recognized by mouse *anti-H. pylori* sera (data not shown).The ELISA results showed that there were significantly raised serum IgG and intestinal SIgA antibody levels in NZ-Δsp-*napA* group and NZ-Δsp-*napA*+*ltB* group on day 7 post vaccination, compared with the control groups. The NZ-Δsp-*napA*+*ltB* group had significantly higher SIgA level than the NZ-Δsp-*napA* group (Figure 3). The findings indicate that LtB can increase local secretory IgA rather than systematic IgG level in the vaccine-induced humoral immune response.

### 3.3. Splenocyte Cultivation and Cytokine Assessment

**Figure 4:**
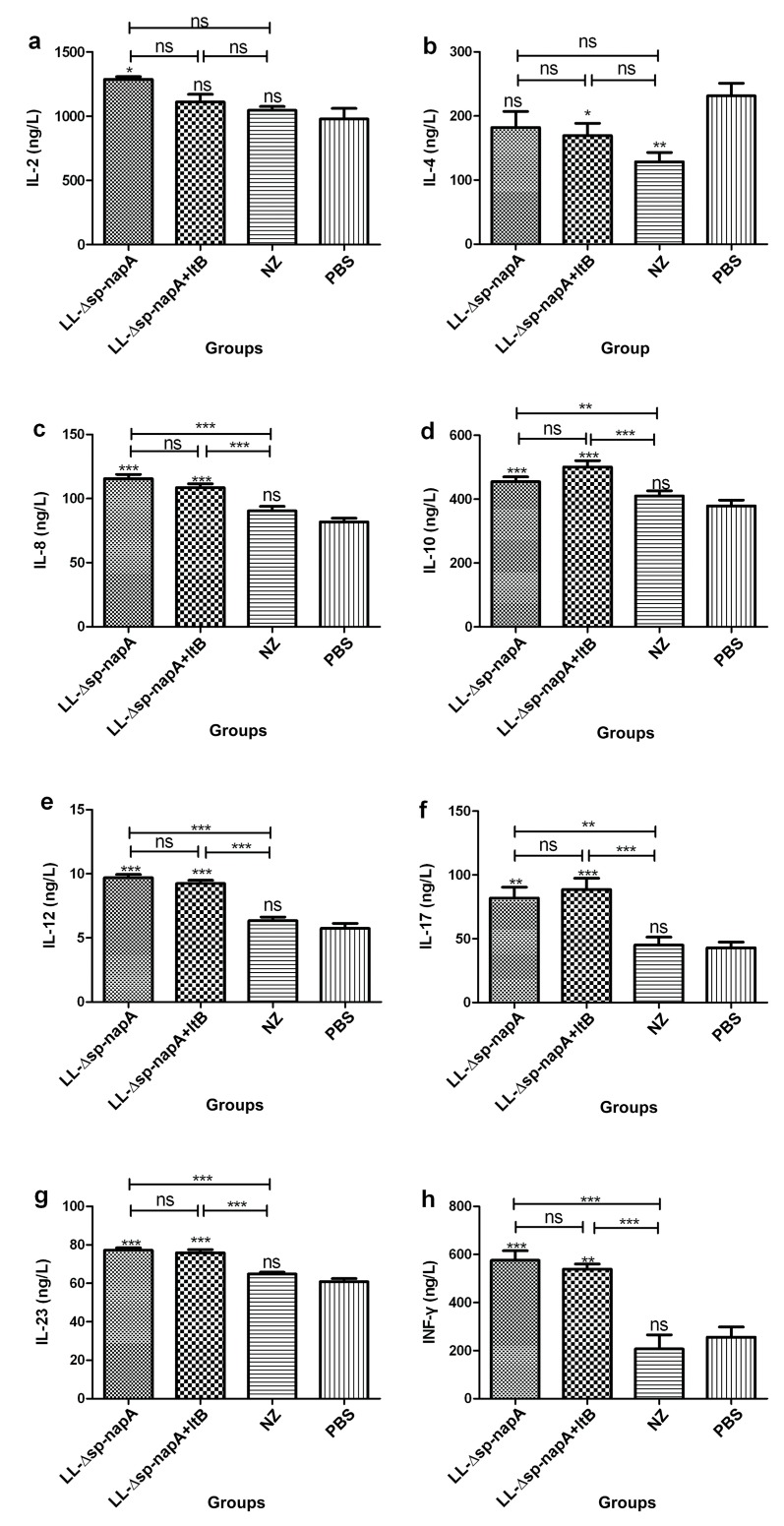
Cytokine expression levels of in vitro cultivated splenocytes (**a**–**h**). Splenocytes were separated from the mice one weeks post immunization, and cultivated and stimulated with *H. pylori* cellular lysates for 3 days. The cytokines in the culture supernatant was measured using ELISA with three replicates. Data were expressed as mean ± SEM. Error bars indicate standard deviations. NZ-*Δsp-napA*, NZ-*Δsp-napA+ltB*, NZ and PBS groups, *n* = 6 each group; ns: no significance, *p* ≥ 0.05; *, **, ***: *p* < 0.05, *p* < 0.01, *p* < 0.001, respectively, compared with the PBS or designated group.

The splenocytes of the mice were cultured and stimulated with *H. pylori* cell lysates post immunization. On day 3 of the cultivation, the culture supernatant was separated, and tested for IL-2, IL-4, IL-8, IL-10, IL-12, IL-17, IL-23, and INF-γ using ELISA. Both NZ-Δsp-*napA* and NZ-Δsp-*napA*+*ltB* groups had significantly enhanced IL-2, IL-8, IL-10, IL-12, IL-17, IL-23, and INF-γ levels (*p* < 0.05), compared with the PBS group (Figure 4). No significant difference in the cytokine IL-4 level was detected between the NZ-Δsp-*napA* and PBS groups, while the NZ-Δsp-*napA*+*ltB* group had significantly lowered IL-4 concentration than the PBS group (*p* < 0.05). The increased IL-17 and IL-23 production in the NZ-Δsp-*napA* group and NZ-Δsp-*napA*+*ltB* group indicates a polarized Th17 response, while the markedly elevated IL-12 and INF-γ production supports a Th1 bias response. These data revealed that gavages with NZ3900/pNZ-Δsp-*napA* alone or in combination with NZ3900/pNZ-sp-*ltB* were able to elevate Th1/Th17 responses, and the combined use of the two strains could significantly inhibit the IL-4 production, suggesting that LtB could promote the NapA-evoked Th1 polarization by inhibition of the Th2 cytokine production.

### 3.4. H. pylori Challenge and Immune Protection

**Figure 5:**
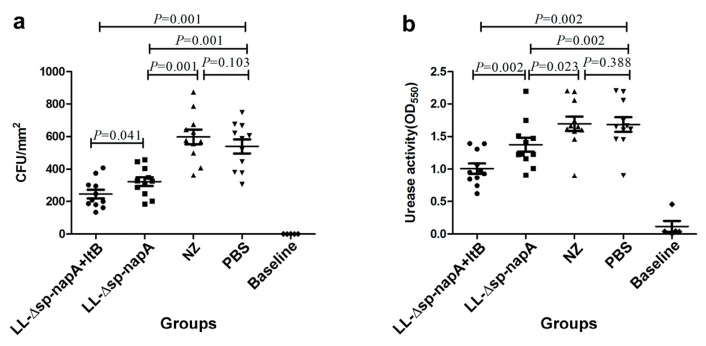
Assessment of *H. pylori* colonization and histological change in the stomachs. The gastric tissues of mice were examined in the first week after *H. pylori* challenges. (**a**) Gastric *H. pylori* colonization levels assayed via bacterial cultivation. (**b**) Gastric bacterial burden evaluated using the urease tests. (**c**) Comparison of the groups on severity of gastric inflammatory changes. Data were expressed as mean ± SEM. Error bars indicate standard deviations. NZ-*Δsp-napA*, NZ-*Δsp-napA+ltB*, NZ and PBS groups (*n* = 11 each) were gavages with NZ3900/pNZ-Δsp-*napA*, mixture of NZ3900/pNZ-Δsp-*napA* and NZ3900/pNZ-sp-*ltB*, NZ3900/pNZ8110 and PBS, respectively, and challenged with *H. pylori*. No gavages were performed on Baseline group (*n* = 10). Ns: no significance; *: *p* < 0.05, Fisher’s LSD test, compared with the PBS or designated group.

After the NZ-Δsp-*napA*, NZ-Δsp-*napA*+*ltB*, NZ and PBS groups were challenged with *H. pylori*, stomach biopsies were performed by urease activity tests and *H. pylori* cultivation. As observed, there were significantly lowered urease activity and bacterial colonization levels in NZ-Δsp-*napA* and NZ-Δsp-*napA*+*ltB* groups than in NZ and PBS groups, while the urease activity was even weaker in NZ-Δsp-*napA*+*ltB* group than NZ-Δsp-*napA* group. The findings indicated the significant protective efficacy of the oral delivery of NapA and the immunologic enhancement effect of LtB as a mucosal adjuvant (Figure 5a,b).

### 3.5. Gastric Histological Examination

**Figure 6:**
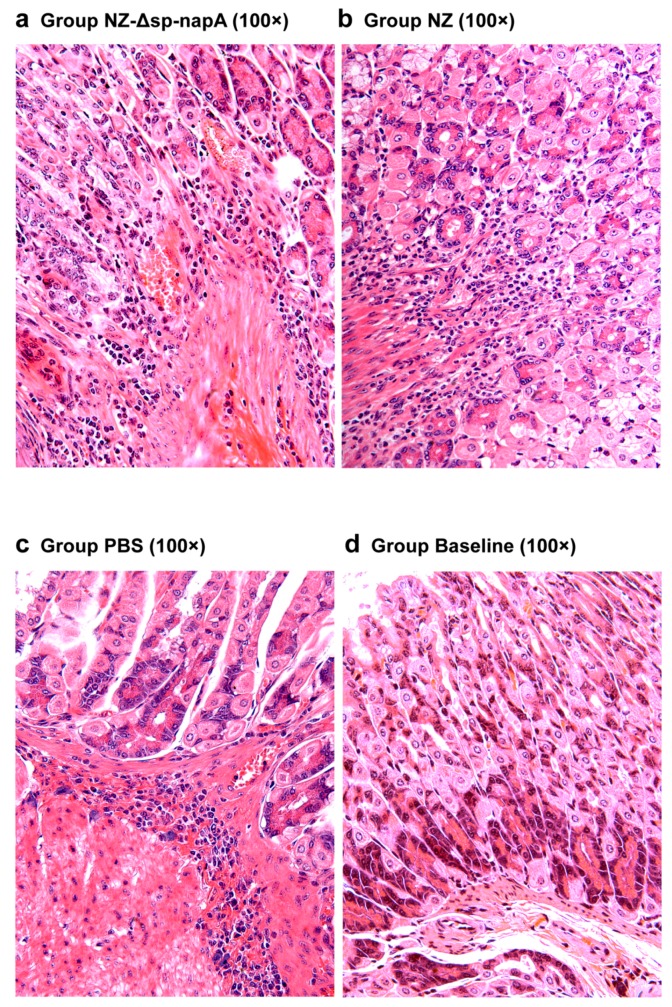
Histological examination of gastric tissues of the LtB-untreated mice. NZ-*Δsp-napA* (**a**), NZ (**b**) and PBS (**c**) groups (*n* = 11 each) were gavaged with NZ3900/pNZ-Δsp-*napA*, NZ3900/pNZ8110 and PBS, respectively, prior to *H. pylori* challenges. No gavages were given to the Baseline group (*n* = 10) (**d**). The gastric tissues were sampled from the mice 1 week after the challenges and examined via paraffin section and hematoxylin-eosin staining. The mice of all the *H. pylori*-challenged groups had gastric inflammatory responses characterized by the infiltration of lymphocytes, neutrophils and microphages, abscess, and congestion in gastric mucosa and submucosa.

**Figure 7:**
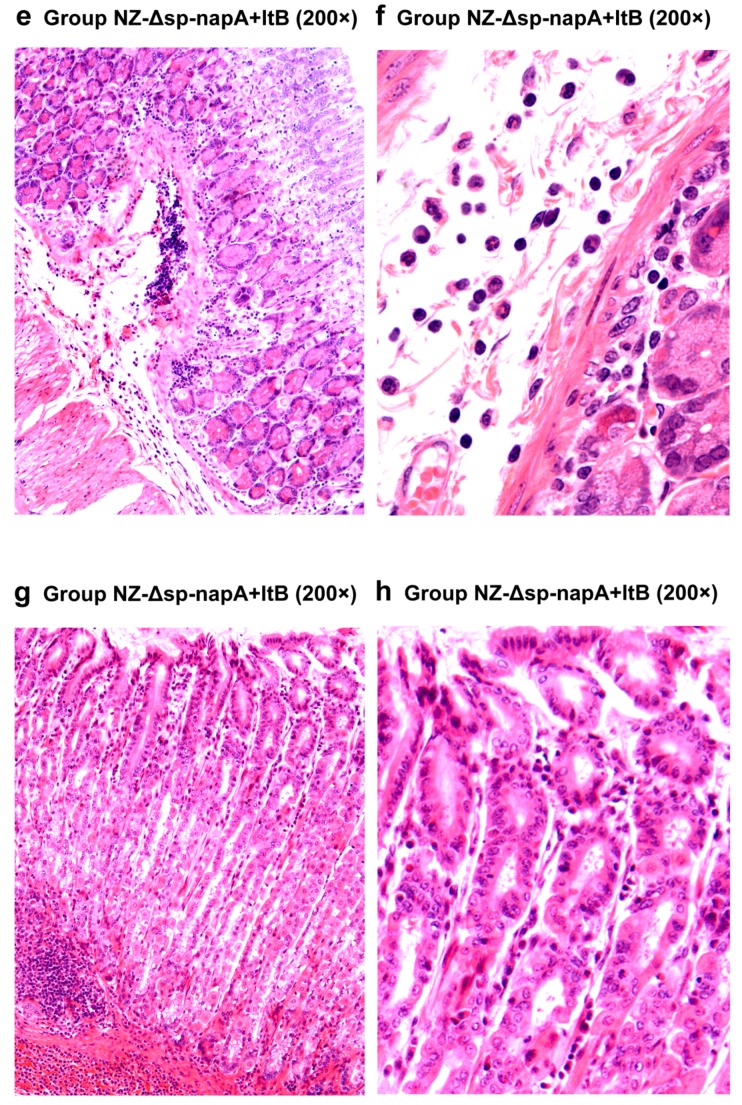
Histological examination of gastric tissues of the NapA+LtB immunized mice (**e**–**h**). NZ-*Δsp-napA+ltB* group (*n* = 11) were gavaged with mixture of NZ3900/pNZ-Δsp-*napA* and -sp-*ltB* and challenged with *H. pylori*. The gastric tissues were sampled from the mice 1 week after the challenges and examined via paraffin section and hematoxylin-eosin staining. The gastric inflammatory changes had higher severity in the NapA+LtB immunized mice than the others, characterized by more serious infiltration of leukocytes, abscess and erosion in gastric mucosa and submucosa.

**Figure 8:**
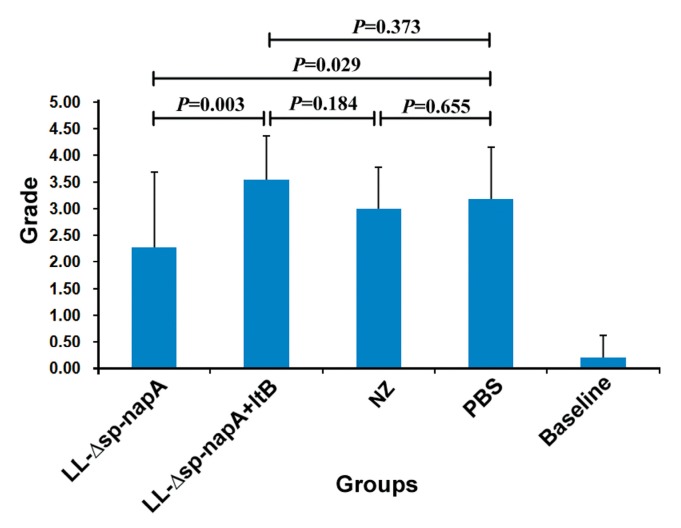
Comparison of the groups on severity of gastric inflammatory changes. Gastric inflammatory changes of the mice were graded using the reported method [25,31]. Data were expressed as mean ± SEM. Error bars indicate standard deviations. NZ-*Δsp-napA*, NZ-*Δsp-napA+ltB*, NZ and PBS groups (*n* = 11 each) were gavages with NZ3900/pNZ-Δsp-*napA*, mixture of NZ3900/pNZ-Δsp-*napA* and NZ3900/pNZ-sp-*ltB*, NZ3900/pNZ8110 and PBS, respectively, and challenged with *H. pylori*. No gavages were performed on Baseline group (*n* = 10). Fisher’s LSD test, compared with the PBS or designated group.

**Figure 9:**
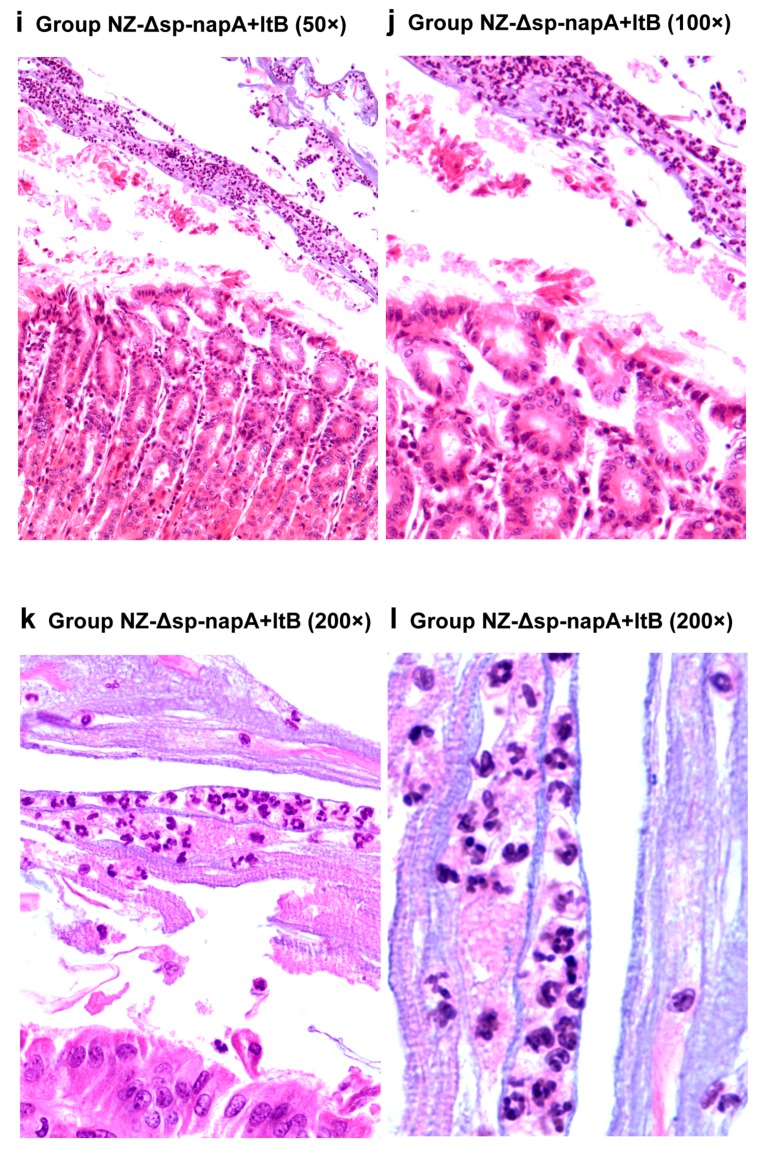
Distribution of leukocytes in gastric mucus of the NapA+LtB immunized mice (**i**–**l**). NZ-*Δsp-napA+ltB* group (*n* = 11) were challenged with *H. pylori* following gavages with mixture of NZ3900/pNZ-Δsp-*napA* and -sp-*ltB*. The gastric tissues were sampled from the mice 1 week after the challenges and examined via paraffin section and hematoxylin-eosin staining. Leukocytes including neutrophils, macrophages and lymphocytes accumulated in the deep and superficial mucosa and gastric mucus.

The mice of all the *H. pylori*-challenged groups had gastric inflammatory responses, characterized by abscess, erosion, infiltration of neutrophils, lymphocytes, monocytes and microphages in gastric mucosa and submucosa (Figure 6 and Figure 7). According to the gastritis grading scores, NZ-Δsp-*napA* group displayed the lowest inflammation level among the *H. pylori* challenged groups, whereas the NapA+LtB immunized mice had significantly higher inflammatory lesion than the mice treated with NapA alone (*p* < 0.05) (Figure 8). Notably, it was observed for the first time that a large amount of neutrophils, lymphocytes and microphages leaked or migrated into the mucus layer in certain mice of NZ-Δsp-*napA*+*ltB* group (5/11) (Figure 9) and NZ-Δsp-*napA* group (1/11). In the lamina propria, leukocytes, mainly neutrophils and macrophages, accumulated near to gastric muscularis mucosa and superficial epithelia, respectively, to form abscesses and epithelial erosions. In the tissue connecting the muscularis mucosa and the superficial epithelia, the leukocytes frequently appeared in lines, seemed to be migrating from the deep to the superficial mucosa (Figure 7g,h). No significant difference was detected in inflammation levels between NZ and PBS groups (*p* > 0.05) and no leukocyte accumulation in the gastric mucus in NZ and PBS groups. These findings prove that LtB significantly aggravates inflammatory responses and immunologic injury in the mouse stomachs when enhancing NapA-induced immune protection.

## 4. Discussion

In the present animal experiments, the mice immunized using NapA with/without LtB had significantly enhanced serum IgG and fecal SIgA levels, and decreased bacterial colonization in the stomach, demonstrating the immune protective efficacy of this engineered strain. The elevated IL-8, IL-10, IL-12, IL-17, IL-23 and INF-γ production in the immunized groups revealed Th1/Th17 biased immune responses. These findings indicated the association between the Th1/Th17 polarization and the NapA-induced protective efficacy, supporting that the anti-*H. pylori* immune protection can be mediated by Th1/Th17 responses [10]. Compared with using NapA alone, additional administration of LtB to mice was capable of reducing *H. pylori* colonization, enhancing SIgA secretion level, which confirmed the adjuvant activity of LtB for mucosal vaccination, showing accordance to certain previous studies [15].

However, despite the observation of LtB potent adjuvanticity, the mechanism beneath LtB enhancement of the protective effect remains unclear [32]. LtB could bind to the GM1 ganglioside receptor, affect the turnover, development and antigen presentation of dendritic cells, clustering of lymphocytes, and B cell uptaking antigens [14,33]. Most reports show LtB/mutant Lt-assisted mucosal vaccination may produce enhanced Th1, Th1/Th2, Th1/Th17, or Th1/Th2/Th17 type immune responses, while Th1/Th17 bias reaction is thought to be associated with the *anti-H. pylori* immune protection [34,35,36]. Recent study observed that LtB elevated transcription of TNFα, IL-12, IL-1αβ, IL-2, IL-6 and chemokines CXCL1, CXCL2, CXCL3, CCL2, CCL3, etc, in dentritic cells [37]. IL-12 can promote differentiation of Th0 into Th1 while IL-1β and IL-6 prompt development of Th0 to Th17. In the present study, the IL-4 level was significantly lowered in NapA+LtB treated mice, but not in NapA immunized mice, in comparison with the unimmunized controls. The inhibition of IL-4 production is capable of promoting Th1 bias differentiation, which may contribute to the protective immunity. Nevertheless, there is no significant increase in IL-8, IL-10, IL-12, IL-17, IL-23, and INF-γ production in NapA+LtB group, compared with NapA group, indicating that although both LtB and NapA can promote Th1/Th17 polarized response, their comprehensive effect might be unable to further increase these cytokines levels. Notably, the findings suggest that LtB enhancement of NapA-induced protective efficacy might be mediated by more highly promoting the Th1/Th17 responses.

By histological examination, this study observed the NapA immunized mice had significantly lower severity of gastritis than the controls treated with PBS. The reasons for this may include that the positive effect of NapA on inflammatory responses was covered up by the negative effect of the bacterial reduction. Notably, NZ-Δsp-*napA*+*ltB* group showed much more severe gastritis than NZ-Δsp-*napA* group. Moreover, accompanying the serious inflammatory changes as abscesses, erosions, leukocyte infiltration in the gastric mucosa and submucosa, accumulated leukocytes, including neutrophiles, microphages, and lymphocytes, were found for the first time in the gastric mucus in NZ-Δsp-*napA* group (1/11) and NZ-Δsp-*napA*+*ltB* (5/11) group. The appearance of leukocytes in the mucus layer was observed more frequently in the NapA+LtB immunized mice than the mice treated with NapA only, which indicated that LtB could exacerbate the gastric inflammatory injury, especially by damaging the epithelial barrier.

**Figure 10:**
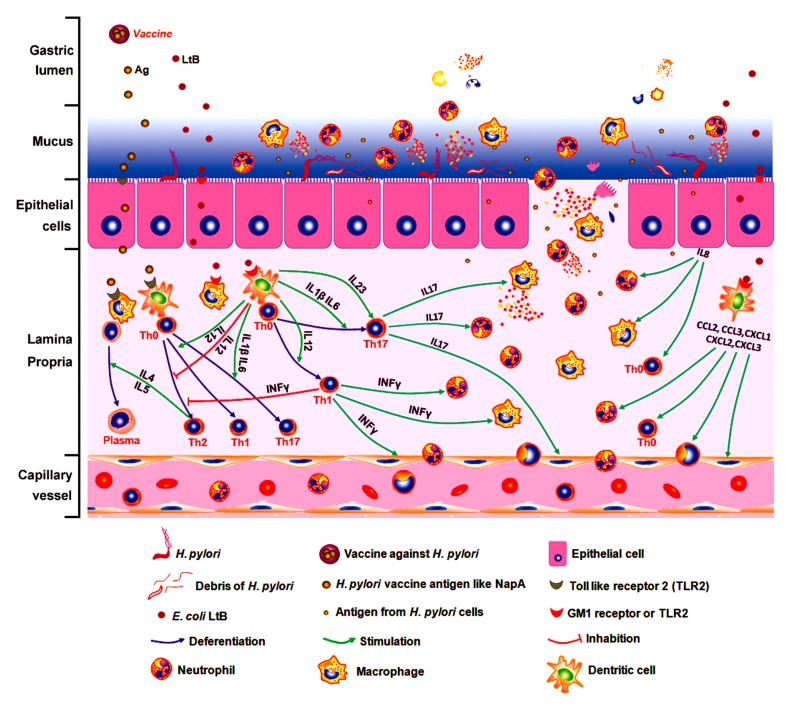
A novel explanation for LtB-adjuvanted oral vaccine-induced immune protection against *H. pylori.* To unveil the mystery of the vaccine-induced cellular immunity destroying *H. pylori* in gastric mucus, we proposed that the LtB-adjuvanted protective immune effect against *H. pylori* involves a sequential process of LtB-aggravated inflammatory response, leukocytes accumulation and degranulation, mucosal injury, leukocytes’ leaking into the mucus layer and killing the microbes by innate with/without acquired immunity. As the most significant observation of this study, leukocytes’ leakage into the mucus is the key to explain the mechanism of the protection.

However, the appearance of the leukocytes in the mucus suggests a possible crucial mechanism for the host to destroy the mucosal non-invasive microorganism with cellular immunity. In the NZ-Δsp-*napA*+*ltB* group, LtB might invade gastric mucosal propria via binding to the GM1 or TLR2 receptor of gastric epithelial cells. This GM1 receptor distributes in almost all the somatic cells. Stimulated by LtB, dentritic cells increased production of TNF-α, IL-1αβ, IL-2, IL-6, IL-12, CXCL1, CXCL2, CXCL3, CCL2, CCL3, CCL4, and CXCL16 [37]. These cytokines and chemokines can affect vascular and lymphatic endothelial cells, activate and accumulate more leukocytes to submucosa and mucosa. LtB, TNF-α and *H. pylori* components like NapA can stimulate gastric epithelial cells to produce chemokines like IL-8 [38,39]. Under the chemotaxis, leukocytes like neutrophils migrate from the deep mucosa to the superficial mucosa (Figure 7g,h). During the process, the activated neutrophils and microphages start degranulation when contacting foreign components like LtB and NapA, and form abscesses and epithelial erosions. Then the leukocytes accumulated near the epithelium may have chance to leak or migrate to the mucus layer via the epithelial injury, approach *H. pylori* and destroy these mucus-colonizing bacteria, as shown in Figure 10 [34,40]. In short, we thought the LtB-adjuvanted protective immune effect against *H. pylori* involves a sequential process of LtB-aggravated inflammatory response, leukocytes accumulation and degranulation, mucosal injury, or leukocytes’ leaking into the mucus layer and killing the microbes with/without acquired specific immunity [41,42,43]. This novel viewpoint might thresh light on the mechanism beneath the cellular immune protection against such organisms as *H. pylori,* ETEC and some *Shigella* species, which are predominantly non-invasive, commonly colonize mucosal surface.

Based on the viewpoint, certain phenomena reported before can be explained. (a) Oral gavages of mice with mucosal adjuvant, cholera toxin B subunit (CTB), but without any *H. pylori* antigens, could still elicit strong anti-*H. pylori* protective immunity [6]. Now, it can be explained by that CTB, which has the similar GM1 receptor-binding activity and molecular characters as LtB, could take the protective effect by the similar mechanism as LtB, which could be independent of the *H. pylori*-specific immunity. (b) Gram-positive enhancer matrix particles (GEM), a mucosal adjuvant produced by *L. lacits*, could evoke Th1/Th17 immune response and reduced *H. pylori* colonization in mice when using alone by oral gavage [41]. This observation also shows that the non-specific protective effect against *H. pylori* may most likely mediated by innate immunity, which coincides with our viewpoint.

Nevertheless, the severe informatory injury linked to LtB probably is a serious problem for the safety of this adjuvant, which might have been overlooked for long term. LtB was considered to be a so-called non-toxic subunit of enterotoxin, to a large extent due to it being free of ADP-ribosylation activity [18,40]. However, recent studies showed that LtB can not only have affinity to the ganglioside receptor, but also bind to the blood group antigens, milk oligosaccharides, and certain gut commensal bacteria [40]. These findings indicate that LtB can play roles in the immunization process by much more ways than binding to the GM1 receptor. Until now, most studies have focused on the adjuvantivity of LtB to enhance immunity, but rarely on the possible immunopathological injury. Factually, immune protection efficacy should include reduction of both infection and histological injury. Significantly, the present study indicates for the first time the urgent necessity of further investigation of the potential hazards of using LtB in vaccination to avoid tremendous social consequences like those reported [44,45]. In particular, clinical trials have been conducted for LtB-UreB-based anti-*H. pylori* vaccines since 2005, though even today no commercial products have resulted from this [46]. 

## 5. Conclusions

In conclusion, this study observed the distribution of leukocytes in the gastric mucus of the LtB-adjuvanted immunized mice for the first time, and thereby proposed the novel explanation for the mechanism of the immune protection. Meanwhile, the findings indicate that LtB can enhance oral vaccine-induced protection via aggravating mucosal inflammatory injury and leukocyte leak, thereby suggesting that this is a serious safety problem on related to the application of LtB as an adjuvant in humans. This report may have considerable impact on future research on mucosal immunology.

## Supplementary Materials

The following are available online at https://www.mdpi.com/2073-4409/8/9/982/s1. Table S1: Plasmid vectors and bacterial strains used herein.
