# Supplementary material for: E. coli Enterotoxin LtB Enhances Vaccine-Induced Anti-H. pylori Protection by Promoting Leukocyte Migration into Gastric Mucus via Inflammatory Lesions
Source: Cells. 2019 Aug 27;8(9):982. doi: 10.3390/cells8090982 (PMC6770474; doi:10.3390/cells8090982)
Supplement: Supplementary file 1 [file cells-08-00982-s001.pdf]

Supplementary Materials

Table S1. Plasmid vectors and bacterial strains used herein.

	Profiles	Reference
Plasmids		
pNZ8110	cm^r, *E. coli*-*L. lactis* shuttle vector with the usp45 signal sequence and the *nisA* promoter	NIZO Food Research, Netherlands
pNZ-Δsp-*napA*	cm^r, pNZ8110 carrying *napA* and deleting the usp45 signal sequence	CGMCC* No.14933
pMAL-c2x-*linker*-*napA*	ap^r, pMAL-c2x (NEB, England) carrying a linker (5'-GGAGGCGGT-3') and *napA* gene	Previous study
Strains		
L. lactis NZ3900	Derivatives of *L. lactis* subsp. cremoris MG1363, $lacF^-$, pepN::nisRnisK, food grade	NIZO Food Research, Netherlands
L. lactis NZ3900 -pNZ-sp-*ltB*	*L. lactis* NZ3900 harboring pNZ-sp-*ltB*	Previous study
L. lactis NZ3900 -pNZ-Δsp-*napA*	*L. lactis* NZ3900 harboring pNZ-Δsp-*napA*	CGMCC* No.14933
E. coli TB1 -pMAL-c2x-linker-*napA*	*E. coli* TB1 harboring pMAL-c2x-linker -*napA*, amp^r	Previous study
H. pylori MEL-Hp27	*cagA+, vacA+*, isolated from a Chinese patient with chronic atrophy gastritis	CGMCC* No.1338
H. pylori 11637	*cagA+, vacA+*, type strain	NCTC11637

*CGMCC, China General Microbiological Culture Collection Center
